# Acanthosis Nigricans Associated with an Adrenocortical Tumor in a Pediatric Patient

**DOI:** 10.1155/2013/174593

**Published:** 2013-05-30

**Authors:** Elizabeth Isaacoff, Filippina Filia Dimitriadi, Frank Barrows, Bruce Pawel, Peter Mattei, Sogol Mostoufi-Moab

**Affiliations:** ^1^Department of Pediatrics, The Children's Hospital of Philadelphia, 34th Street and Civic Center Boulevard, 3535 Market Street, Room 1562, Philadelphia, PA 19104, USA; ^2^Division of Pediatric Endocrinology, St. Christopher's Hospital for Children, 3601 A. Street, Philadelphia, PA 19134, USA; ^3^Division of Pediatric Endocrinology, Monmouth Medical Children's Hospital, 180 Avenue at the Commons, Suite 7B, Shrewsbury, NJ 07702, USA; ^4^Department of Pathology, The Children's Hospital of Philadelphia, 34th Street and Civic Center Boulevard, Philadelphia, PA 19104, USA; ^5^Department of General Surgery, The Children's Hospital of Philadelphia, 34th Street and Civic Center Boulevard, Philadelphia, PA 19104, USA

## Abstract

Malignant acanthosis nigricans (AN) is a rare paraneoplastic syndrome seen primarily in adults with an underlying diagnosis of gastrointestinal adenocarcinoma. Malignant AN is characterized by hyperpigmentation and velvety hyperplasia of the epidermis. This condition is generally not associated with tumors in pediatric populations or in the adrenal gland. We present a case of malignant AN in a pediatric patient with a nonmalignant, functional adrenocortical tumor.

## 1. Introduction 

Childhood adrenocortical tumors (ACTs) generally present during the first 5 years of life, with a second, smaller peak noted during adolescence [1–4]. Majority of these tumors come to medical attention subsequent to overproduction of adrenal cortical hormones. Virilization is the most common presenting sign (84.3%), either alone (55.1%) or in combination with overproduction of other adrenal hormones including aldosterone or glucocorticoids (29.2%), followed by 5.5% with isolated Cushing's syndrome, and 10.2% with nonfunctional tumors [4].

The diagnosis of ACTs in pediatric patients is generally made within 5–8 months of the first presenting signs and symptoms [2, 4, 5]; however, one-third of pediatric patients have either unresectable or metastatic disease at the time of diagnosis. The remaining two-thirds have disease confined to the adrenals [4]. An elevated blood or urine concentration of adrenocortical hormones and a suprarenal mass generally suggest a preoperative diagnosis of ACT. Imaging studies are necessary for sufficient staging and surgery planning [6]. Surgery is the best treatment plan for those with ACTs, and cisplatin-based chemotherapy with mitotane is indicated for metastatic disease or when complete resection is not possible at presentation [6]. In children with localized ACTs, tumor weight ≤200 g, virilization alone, stage I disease, absence of spillage during surgery, and age ≤3 years are considered important favorable prognostic indicators [4]. 

Malignant acanthosis nigricans (AN) is a rare paraneoplastic syndrome seen mainly in adults with adenocarcinomas, primarily of gastric origin [7, 8]. Malignant AN appears concurrently at the time of tumor diagnosis in 61.3%, but may also appear before (17.6%) or after (21.1%) cancer diagnosis [9]. Malignant AN is usually not a recognized paraneoplastic finding associated with pediatric tumors. Here, we report the first case of malignant AN in a pediatric patient with a nonmalignant, functional ACT at the time of tumor diagnosis. 

## 2. Case Presentation

A previously healthy 33-month-old Caucasian male presented with a three-month history of rapid virilization and a neck rash. Height and weight remained unchanged over the preceding months at the 50th percentile. Blood pressure at the time of diagnosis was 123/65 mmHg and heart rate 112 bpm. Physical examination was notable for coarse facial features, facial acne, anterior and posterior cervical acanthosis nigricans, Tanner 2 pubic hair, and pubertal phallus (8 cm in length and 2 cm in diameter), but prepubertal testes at 3 mL volume bilaterally. Laboratory evaluation revealed an elevated nonfasting insulin level with normal values for glucose, random cortisol 19.6 mcg/dL (normal range 9–22 mcg/dL), ACTH 8.35 pg/mL (normal range 5–46 pg/mL), and hemoglobin A1C 4.7% (normal range 3.8–5.9%). Abnormal laboratory findings for age included elevated testosterone, androstenedione, dehydroepiandrosterone sulfate (DHEAS), all within Tanner 2-3 range. IGF-I level was increased for age. Gonadotropins were Tanner stage 1, confirming a peripheral source for androgen production. Serum electrolytes, beta-HCG, and alpha-fetoprotein levels were all within normal limits.  Table 1 is a summary of pertinent laboratory results.

**Figure 1:**
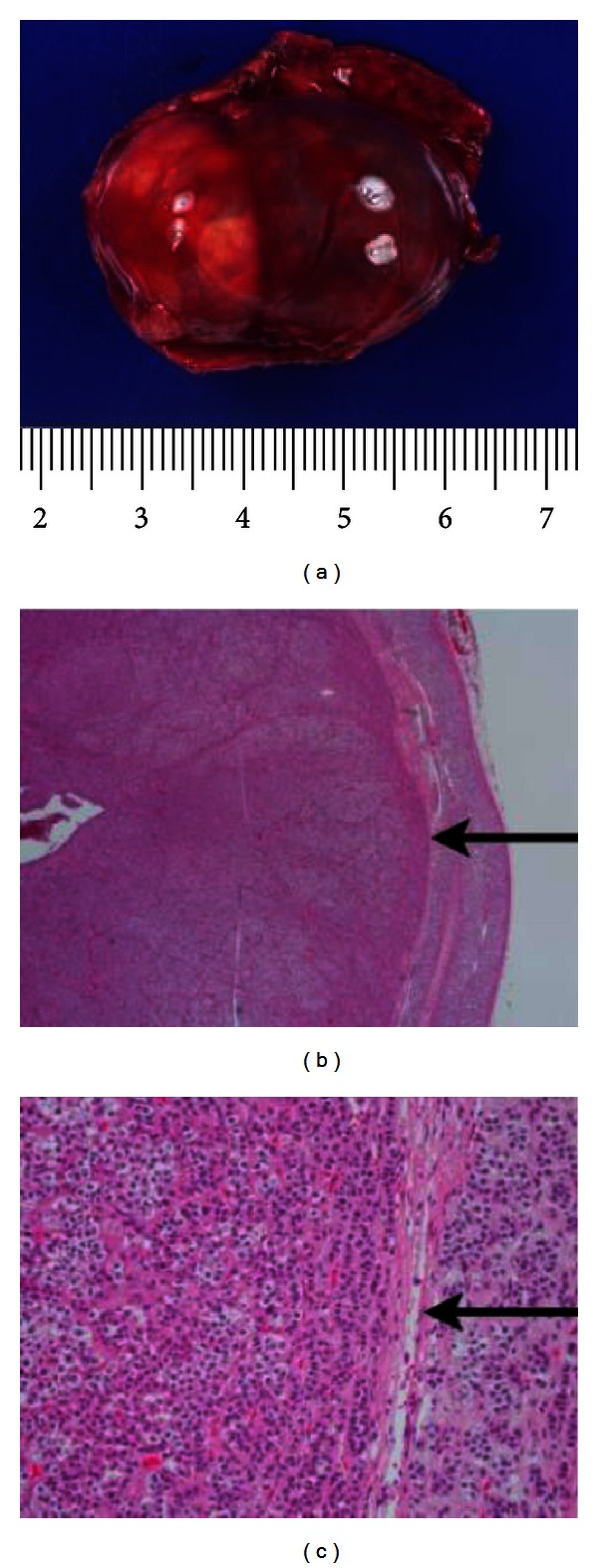
(a) Gross photograph of resected adrenal mass. (b) Low power photomicrograph of adrenocortical tumor. The tumor is encapsulated (arrow) and has a pushing border, with compressed normal adrenal cortex on the right. (H&E, 20x). (c) High power photomicrograph of adrenocortical tumor. Arrow is pointing to a thin fibrous capsule. The tumor has similar cytologic features to the normal adrenal seen on the right. Features that have been associated with malignancy (capsular invasion, necrosis, and increased mitotic activity) are not present (H&E, 200x).

MRI of the abdomen revealed a 2.3 × 3 cm mass within the right adrenal cortex, absent retroperitoneal lymphadenopathy, and lack of tumor thrombus within the IVC. The patient underwent a successful right adrenalectomy and retroperitoneal lymph node dissection without any complications. Postoperative screening morning cortisol was undetectable (<1.0 mcg/dL), indicating suppression of contralateral unaffected adrenal gland. He was treated with stress-dose hydrocortisone and subsequently transitioned to and slowly weaned off of physiologic replacement postoperatively. Follow-up random cortisol level one year postoperatively was normal at 7.1 mcg/dL (normal range 1.5–9.0 mcg/dL). Pathology review revealed a 12.5-gram encapsulated tumor without hemorrhage, necrosis, or capsular invasion (Figure 1). Tumor cells exhibited rare mitotic figures, favoring the diagnosis of a non-malignant adrenal cortical tumor. He demonstrated marked clinical improvement of all symptoms, including normalization of all laboratory values three weeks after surgery and complete resolution of the acanthosis nigricans three months postoperatively. 

## 3. Discussion

ACT peaks during the first and fourth decades of life [10]. The incidence of ACT varies internationally, with particularly high rates noted in southern Brazil, where the incidence is approximately 10–15 times that observed in the USA [6]. Predisposing genetic factors may be responsible for this increased incidence [6]. ACT in children is exceptionally rare, composing only 0.2% of pediatric cancers [11]. Only 25 new cases are expected to occur annually in the USA, for an estimated annual incidence of 0.2–0.3 cases per million [11]. 

Acanthosis nigricans (AN), a common cutaneous finding, is characterized by hyperpigmentation and velvety hyperplasia of the epidermis [12]. In general, it affects flexural areas including the neck, antecubital, and, the popliteal fossa [12]. Benign AN usually presents between birth and puberty and can have a possible genetic component [8]. Benign AN often occurs in individuals exhibiting insulin resistance, such as patients with a diagnosis of diabetes mellitus, obesity, and polycystic ovarian syndrome [12]. Although benign forms of AN are relatively common, malignant AN can occur as a rare paraneoplastic syndrome with approximately 1,000 reported worldwide cases [13]. It is most often seen in adults with an underlying diagnosis of gastrointestinal adenocarcinoma [7, 8]. Malignant AN tends to worsen with progression of the neoplasm, lessen with treatment, and return with tumor recurrence and/or metastases [8]. Malignant AN is extremely uncommon in pediatric tumors [14, 15] with only one reported pediatric case of malignant AN associated with a malignant adrenal tumor [16].

The pathophysiology of malignant AN is not well understood. Elevated levels of growth factors released from tumors, such as transforming growth factor alpha (TGF-alpha), can bind to epidermal growth factor receptors (EGFR) in the epidermis and stimulate keratinocyte growth [7, 12, 17]. Malignant AN can be associated with the sign of Leser-Trélat (LT), a rare and sudden onset of multiple benign skin growths originating in keratinocytes (seborrheic keratoses) and associated with an internal malignancy [7, 12]. Biopsy specimens of seborrheic keratoses in a patient with LT, malignant AN, and melanoma revealed elevated levels of urinary TGF-alpha and increased epidermal staining of EGFRs, with decreased levels of both after treatment of the melanoma [17]. Hereditary diseases associated with AN demonstrate activating germ line mutations in the fibroblast growth factor receptor 3 (FGFR3) gene, which increase keratinocyte growth [18–21]. A positive association of FGFR3 expression has been reported in two cases of malignant AN, with expression throughout the acanthotic lesions [22].

Our patient's AN likely resulted from excess glucocorticoid production by the adrenal tumor, albeit not captured in preoperative laboratory studies. His elevated IGF-1 driven by increased adrenal androgens may have also played a role. Contralateral adrenal cortical suppression with undetectable cortisol and suppressed ACTH after surgery points to excess glucocorticoid hormone secretion by the adrenal mass in addition to the noted elevated adrenal androgens. The excess glucocorticoid production likely contributed to glucocorticoid-induced insulin resistance, resulting in AN. This case represents the first reported association of malignant AN with a nonmalignant, functional cortical adrenal mass in a pediatric patient and an important clue to a possible underlying cortical adrenal tumor as a contributing cause for this unusual phenomenon. 

## Figures and Tables

**Table 1:** Pre- and postoperative endocrine laboratory values.

Hormone	Preoperative hormone level	Postoperative hormone level				Normal range
	4/19/2010	5/11/2010	6/30/2010	4/20/2011	9/26/2012	
Glucose (mg/dL)	88	71	127	83	78	74–127
Nonfasting insulin (uU/mL)	43.7	—	30.7	—	—	—
Hemoglobin A1C (%)	4.9	—	—	4.7	4.7	3.8–5.9
Testosterone (ng/dL)	150	<3.0	<3.0	<2.5	<2.5	Tanner 1 range <3–10 Tanner 2 range 18–150
Androstenedione (ng/dL)	72	<10	—	<10	<10	Tanner 1 range <10–17 Tanner 2 range 31–65
Dehydroepiandrosterone sulfate (ug/dL)	73	<10	—	—	—	Range for 1–5 years of age <5–57 Tanner 2 range 42–109
IGF-1/Somatomedin-C serum (ng/mL)	325	—	143	101	111	Range for 3-4 years of age 54–178
FSH (mIU/mL)	0.143	—	—	—	—	Tanner 1 range 0.1–3.0
Luteinizing hormone (mIU/mL)	0.010	—	—	—	—	Tanner 1 range 0.01–0.3
